# Molecular Elucidation of a Urate Oxidase from *Deinococcus radiodurans* for Hyperuricemia and Gout Therapy

**DOI:** 10.3390/ijms22115611

**Published:** 2021-05-25

**Authors:** Yi-Chih Chiu, Ting-Syuan Hsu, Chen-Yu Huang, Chun-Hua Hsu

**Affiliations:** 1 Genome and Systems Biology Degree Program, National Taiwan University and Academia Sinica, Taipei 115024, Taiwan; d04b48005@ntu.edu.tw; 2 Department of Agricultural Chemistry, National Taiwan University, Taipei 10617, Taiwan; 3 Taipei First Girl High School, Taipei 10045, Taiwan; d10631412@gapps.fg.tp.edu.tw (T.-S.H.); d10631318@gapps.fg.tp.edu.tw (C.-Y.H.); 4 Institute of Biochemical Sciences, National Taiwan University, Taipei 10617, Taiwan

**Keywords:** uricase, *Deinococcus radiodurans*, gout therapy

## Abstract

Urate oxidase initiates the uric acid degradation pathways and is extensively used for protein drug development for gout therapy and serum uric acid diagnosis. We first present the biochemical and structural elucidation of a urate oxidase from the extremophile microorganism *Deinococcus radiodurans* (DrUox). From enzyme characterization, DrUox showed optimal catalytic ability at 30 °C and pH 9.0 with high stability under physiological conditions. Only the Mg^2+^ ion moderately elevated its activity, which indicates the characteristic of the cofactor-free urate oxidase family. Of note, DrUox is thermostable in mesophilic conditions. It retains almost 100% activity when incubated at 25 °C and 37 °C for 24 h. In this study, we characterized a thermostable urate oxidase, DrUox with high catalytic efficiency and thermal stability, which strengthens its potential for medical applications.

## 1. Introduction

Urate oxidase (uricase; Uox; EC 1.7.3.3) initiates a series of purine degradation pathways that catalyzes the oxidation of uric acid (UA) independent of a cofactor [1]. In brief, it catalyzes the hydroxylation of UA with O_2_ and H_2_O to obtain hydrogen peroxide (H_2_O_2_) and an unstable product 5-hydroxy-isourate (5-HIU) [2]. 5-HIU can evolve spontaneously to a racemic mixture of allantoin in solution or enzymatically degraded into (S)-allantoin as the end product of purine metabolism [2,3]. For most prokaryotes and eukaryotes, this compound exhibits high aqueous solubility ready for urinary excretion [2,4]. However, in humans and some primates, the urate oxidase has lost functional activity due to the accumulation of nonsense and missense mutations (pseudogenization) within the coding sequence of the *Uox* gene during the evolutionary process [5,6,7]. As a consequence, instead of allantoin, UA becomes the resulting product of purine metabolism in hominoids [7].

In humans, most UA dissolves in the blood, travels to the kidneys, and leaves the body in urine. Elevated UA level in blood may increase the risk of hyperuricemia, which is normally defined as serum UA level > 6.8 mg/dL [8]. Chronic hyperuricemia may lead to symptoms of gout, chronic kidney disease, hypertension, and cardiovascular diseases [9,10]. In the clinical case of chemotherapy, acute hyperuricemia may cause acute kidney injury due to massive tumor cell lysis accompanying the UA burst, called tumor lysis syndrome (TLS) [11].

To address these concerns, xanthine oxidase inhibitors and uricosuric agents have been used to reduce the UA synthesis and inhibit renal tubular reabsorption of UA. However, use of these drugs may result in more renal load in long-term therapies [12]. In recent years, the development of urate oxidase (also known as uricase) has become an alternative urate-lowering therapy to rapidly degrade UA in the human body [13]. Elitek (rasburicase) is the first approved clinical drug for preventing and treating TLS and is a recombinant version of urate oxidase from *Aspergillus flavus* [14,15]. Krystexxa (pegloticase), a hyper-PEGylated pig-baboon chimeric urate oxidase, is another approved drug for treating hyperuricemia in patients with chronic refractory gout [16]. Nevertheless, patient adherence is still poor because of low efficacy, side effects, and cost with the long-term therapies [17]. In addition, urate oxidases from various microorganisms, including *Arthrobacter globiforms* [18], *Bacillus fastidious* [18], and *Candida* sp. [19], have been used as diagnostic tools coupled with the 4-aminoantipyrine peroxidase system. Hence, high thermal stability, long-term storage stability, and the activity of urate oxidase plays a crucial role in medical applications and enzymatic analyses.

Several strategies have been used to modulate the enzyme stability and activity. Structure-based protein engineering of urate oxidases from *A. globiforms* and *Bacillus* sp. TB-90 apparently improved their thermal stabilities by strengthening the inter-subunit interactions via disulfide bridges [20,21]. Molecular engineering via directed evolution improved the catalytic activity of an enzyme, such as urate oxidase from *Bacillus subtilis* [22]. However, their enzymatic activities and thermal tolerance under mesophilic conditions are still limited because of lack of suitable starting materials. Here, we present a thermostable urate oxidase (DrUox) from the extremophile baterium *Deinococcus radiodurans*. The tetrameric DrUox demonstrated high catalytic efficiency and thermostability under mesophilic conditions, and so is a valuable candidate for medical applications.

## 2. Results

### 2.1. Amino Acid Sequence Analysis of DrUox

**Figure 1:**
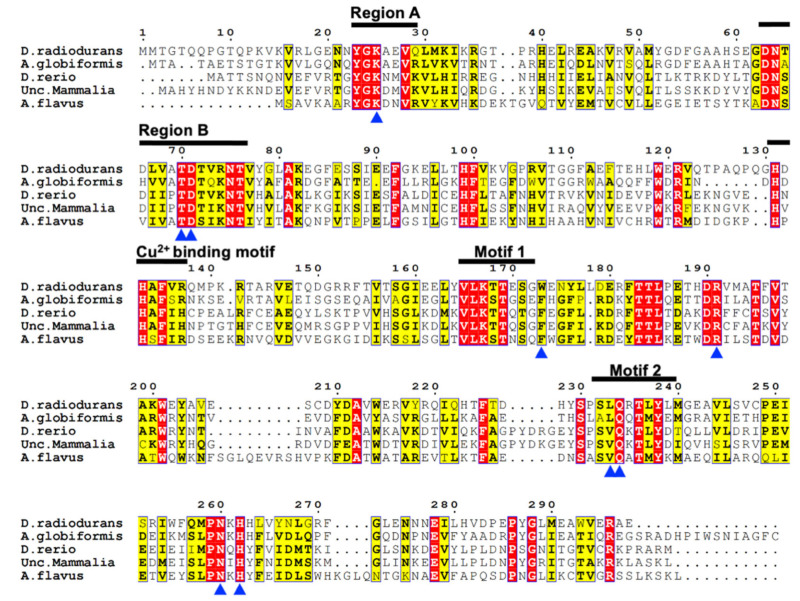
Multiple sequence alignment of DrUox and other relative urate oxidases. Amino acid sequences of DrUox from *D. radiodurans* (WP_010887803.1), AgUox from *A. globiformis* (D0VWQ1.1), DrUox from *D. rerio* (NP_001002332.1), urate oxidase from unclassified mammalia (NP_001011886.1), and AfUox from *A. flavus* (XP_001826198.1) aligned by using Clustal W. Conserved regions of DrUox in comparison other relatives are depicted on the top of the sequence with black lines. Consensus amino acids among these macro domain proteins with similarity score > 0.7 are framed in yellow. Identical amino acids are framed in red. Blue arrowheads on the bottom indicate the key catalytic residues as described in the main text.

The *D. radiodurans* urate oxidase gene (GenBank: AAF10733), named DrUox, was chosen from the NCBI database. The amino acid sequence of DrUox (WP_010887803.1) was aligned with relative urate oxidases (Figure 1). DrUox shared the highest sequence identity with *A. globiforms* urate oxidase (sequence identity: 43%; D0VWQ1.1) and 34% identity with the urate oxidase from *Danio rerio* (NP_001002332.1), 35% identity with the urate oxidase from unclassified mammalia (NP_001011886.1), and 31% identity with the urate oxidase from *A. flavus* (XP_001826198.1).

As described in previous studies, the urate oxidase family commonly contains two conserved regions and two motifs, defined as region A (Y/H-G-K-X-X-V), region B (N-S-X-V/I-V/I-A/P-TD-S/T-X-K-N), motif 1 (V-L-K-T-T-Q-S), and motif 2 (S-P-S-V-Q-K/H/N-T-L-Y), respectively [23,24]. From multiple sequence alignment (Figure 1), DrUox shares identical sequences within region A. However, several amino acids of DrUox sequence within region B are not conserved; in ^64^N-T-D-L-V-A-T-D-T-V-R-^75^N, the ^65^T, ^67^L and ^74^R could be distinct. Motif 1 and 2 in DrUox show nearly consensus patterns; only ^170^E in Motif 1 and ^233^L, ^235^R in Motif 2 are changed. Additionally, some eukaryotic urate oxidases are copper ion-binding proteins [23,25]; the putative cooper ion-binding motif, H-X-H-X-F, was also found in DrUox (Figure 1). Furthermore, the critical catalytic residues of DrUox present in the dimeric interfaces were found to be highly conserved and are marked with a blue arrowhead in Figure 1, which indicates the conserved catalytic mechanism of the urate oxidase family.

### 2.2. Cloning, Expression and Purification of Recombinant DrUox

**Figure 2:**
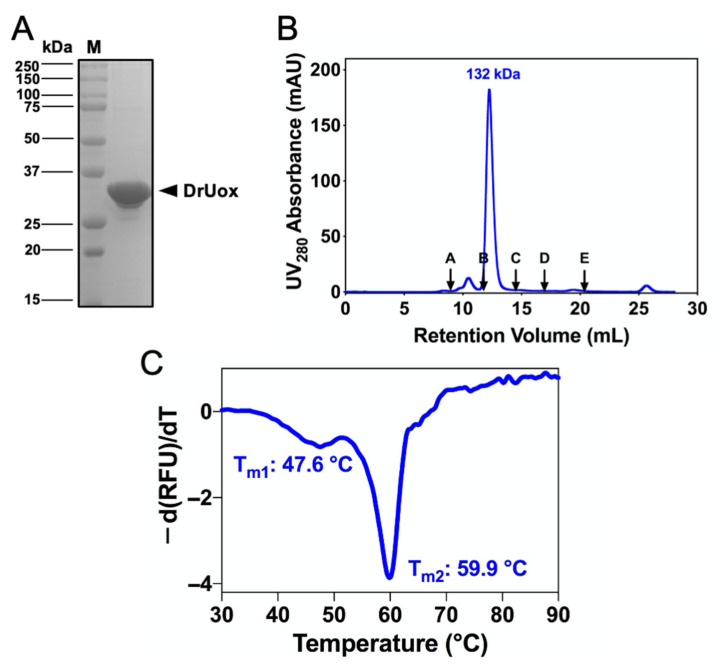
Biochemical and biophysical characterization of urate oxidase from *D. radiodurans* (DrUox). (**A**) SDS-PAGE of purified DrUox, as indicated by black arrow. (**B**) Size-exclusion chromatography of the native DrUox enzyme (blue line). The black arrows indicate the molecular weight (Mw) of protein markers (A: bovine thyroglobulin, 670 kDa; B: bovine gamma globulin, 158 kDa; C: chicken ovalbumin, 44 kDa; D: horse myoglobin, 17 kDa; E: vitamin B12, 1.35 kDa) plotted against elution volume (mL). DrUox exhibits a tetramer form in the native state with the calculated molecular weight of 132 kDa. (**C**) Results of differential scanning fluorimetry of DrUox.

To produce recombinant DrUox, the DrUox gene (Gene ID: DR_1160) was cloned and heterologously expressed in *E. coli* BL21 (DE3) (Supplementary Figures S1 and S2). The N-terminal His_6_-tagged recombinant DrUox was then purified by Ni-NTA affinity chromatography. After purification, the DrUox protomer was 33 kDa, which was >98% pure as determined by SDS-PAGE (Figure 2A). Moreover, size-exclusion chromatography (SEC) revealed that DrUox is a tetrameric protein in the native state (Figure 2B). Thus, DrUox may form a canonical homo-tetramer as do other urate oxidases [26].

The thermal stability of DrUox was then assessed by differential scanning fluorimetry (DSF) (Figure 2C). The DSF curve of DrUox exhibited two distinct transition peaks, which indicates the presence of multiple structural subunits within a protein molecule that unfolds independently. The first peak represented the dissociation of the tetramer into dimers with T_m1_ of 47.6 °C, and the second peak represented the dissociation of the dimer into monomers with T_m2_ of 59.9 °C.

### 2.3. Influence of Temperature and PH on DrUox

**Figure 3:**
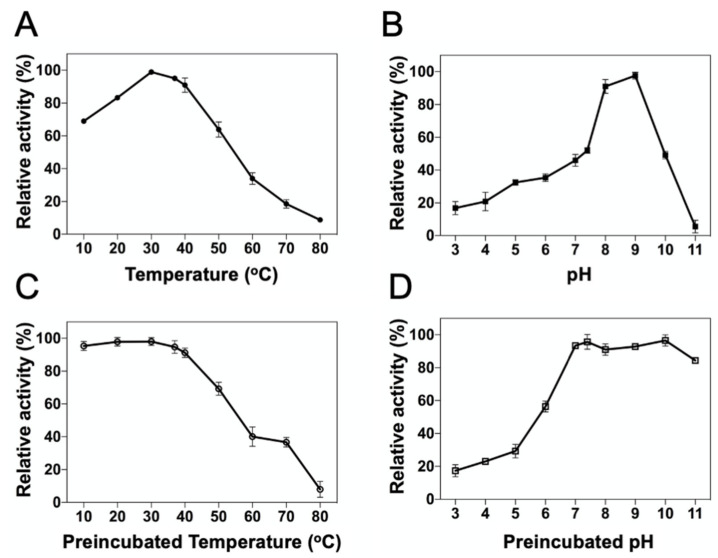
Temperature and pH effect on enzyme activity and stability of DrUox. (**A**) Optimal temperature was evaluated by incubating 0.5 μM DrUox and 500 μM uric acid in a temperature range of 10 °C to 80 °C for 3 min in pH 9.0. (**B**) Optimal pH was evaluated by incubating 0.5 μM DrUox and 500 μM uric acid in a pH range of 3.0 to 11.0 for 3 min at 30 °C. (**C**) Temperature on DrUox stability was determined under the optimal condition after preincubating DrUox at temperatures ranging from 10 °C to 80 °C for 0.5 h. (**D**) pH stability of DrUox was determined under the optimal condition after preincubating DrUox in pH buffers ranging from 3.0 to 11.0 for 24 h at 4 °C. The DrUox activity under different conditions were assayed by monitoring the depletion of uric acid in absorbance at 293 nm.

To investigate the effects of temperature and pH on the catalytic efficiency, the enzyme reaction of DrUox was tested at different pH and temperatures. The optimal temperature of DrUox was determined to be 30 °C (Figure 3A), and it retained more than 80% activity in a temperature range of 20–40 °C. It is noted that DrUox retained more than 90% activity at physiological temperature. In addition, the optimal pH of DrUox was at pH 9.0 (Figure 3B). DrUox retained most activity (>90%) over a pH range of 8.0 to 9.0, which was similar to the urate oxidases from previous studies [14,27]. However, at physiological pH, DrUox retained only 50% activity, which suggests its preference for alkaline conditions. Furthermore, the UA depletion assay was subjected to kinetic monitoring enzyme reaction at various substrate concentrations ranging from normal (100–450 μM) to hyperuricemia (450–600 μM) under optimal (30 °C, pH 9.0) and physiological (37 °C, pH 7.4) conditions. We determined the dependence of initial velocity of an enzyme-catalyzed reaction with initial substrate concentration by using the Michaelis-Menten equation (Supplementary Figure S3) for the apparent kinetic constants as shown in Table 1. Accordingly, DrUox exhibited high catalytic efficiency in the optimal condition, which was 25-fold higher than the physiological condition.

To evaluate the temperature on DrUox stability, thermal inactivation of DrUox was investigated. We preincubated DrUox at various temperatures for short periods, followed by determing the irreversible loss of enzyme activity under the optimal condition (30 °C, pH 9.0). As shown in Figure 3C, DrUox was thermostable below 40 °C, and the residual activity was gradually reduced with increasing temperature. In addition, pH stability of DrUox was estimated with enzyme preincubation in various pH buffer solutions for 24 h at 4 °C, followed by determing the residual DrUox activity under optimal condition. Notably, DrUox retained more than 90% activity in the pH range 7.0–10.0 (Figure 3D) and the retained activity decreased below pH 7.0. Thus, DrUox is thermostable under a mesophilic environment and seems applicable under physiological conditions.

### 2.4. Effect of Metal Ions and Chemicals on DrUox Activity

**Figure 4:**
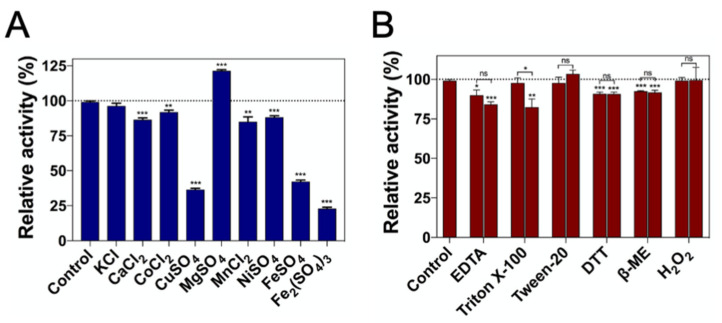
Effect of various metal ions and chemical agents on the enzyme activity of DrUox. (**A**) To evaluate the effect of metal ions on DrUox activity, the purified enzyme was preincubated for 1 h with the following salts: 2 mM each of KCl, CaCl_2_, CoCl_2_, CuSO_4_, MgSO_4_, MnCl_2_, NiSO_4_, FeSO_4_ and Fe_2_(SO_4_)_3_. (**B**) To evaluate the effect of chemical agents, the enzyme was incubated with 10 and 20 mM EDTA, DTT, β-ME; 1% and 2% of Triton X-100 and Tween-20; and 0.2 and 0.5 mM of H_2_O_2_. The enzymatic activity was measured under optimal conditions. The activity without the addition of the above chemicals was designated as 100%. Data are mean ± SEM (*n* = 3) and shown in Supplementary Table S1. * *p* < 0.05, ** *p* < 0.005, *** *p* < 0.0005; ns, not significant, determined by Student’s *t*-test.

We determined the impact of different metal ions on DrUox activity (Figure 4A and Supplementary Table S1). The mechanism of Cu^2+^ on the activity of urate oxidases is intriguing. Some reports have demonstrated that the activity of urate oxidase may be inhibited by Cu^2+^ due to the presence of the Cu^2+^-binding motif (H-X-H-X-F) [23]. In our study, the Cu^2+^-binding motif was conserved within DrUox and led to 60% loss of enzymatic activity upon preincubation with Cu^2+^, which is similar to urate oxidase from *A. globiformis* [23]. Cu^2+^ was shown to inactivate *C. utilis* urate oxidase completely [27]; however, some reports mentioned that urate oxidases from *B. subtilis* and *K. marxianus* (KmUox) were still active in the presence of Cu^2+^ despite the lack of a Cu^2+^-binding motif [28,29]. In addition, urate oxidases appeared to be sensitive to ferrous cation (Fe^2+^) and ferric cation (Fe^3+^). The activity of KmUox is completely inhibited by Fe^2+^ and Fe^3+^ [28], and we found that the activity of DrUox was apparently inhibited by Fe^2+^ and Fe^3+^, with 60% and 80% reduction, respectively. The structural effect of Cu^2+^, Fe^2+^, and Fe^3+^ on DrUox did not change protein oligomerization (Supplementary Figure S4). Other cations were tested: Ca^2+^, Co^2+^, Mn^2+^ and Ni^2+^ had >20% reduction on activity of DrUox, whereas only Mg^2+^ enhanced nearly 20% of DrUox activity. Therefore, DrUox is strongly sensitive to specific metal ions, including Cu^2+^, Fe^2+^, and Fe^3+^, instead of their valence numbers.

We investigated the effect of chemical agents on the activity of DrUox. Surfactants were used to assess the effect of intrinsic enzyme properties, including secondary or tertiary structure changes [30]. Only 2% Triton X-100 had a minor influence on DrUox activity, with a reduction of 17% (Figure 4B). Chelating agent was used to assess the cation dependency of DrUox. The addition of EDTA slightly decreased (10–15%) DrUox activity. Moreover, because the disulfide bonds were not involved in the catalytic mechanism and oligomerization, reducing agents had only little influence on DrUox activity. Furthermore, the active site of urate oxidase was reported to share structural similarities with catalase at the same location occupied by O_2_ then H_2_O and H_2_O_2_ then H_2_O. Hence, O_2_ and H_2_O_2_ may share a common site during urate oxidase catalysis [31]. However, the addition of H_2_O_2_ had no effect on the activity of DrUox.

### 2.5. Effect of Temperature on the Long-Term Stability of DrUox

**Figure 5:**
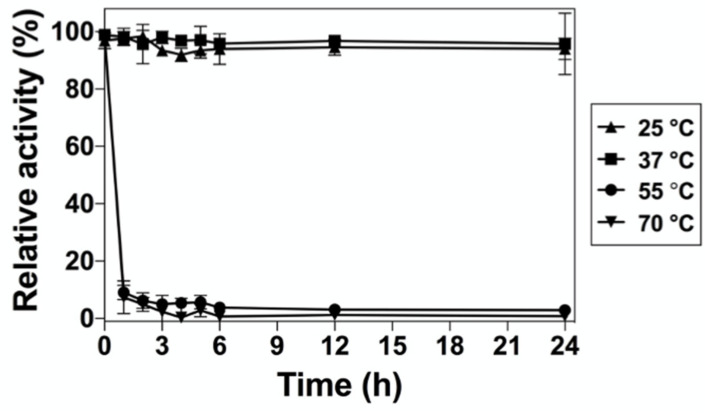
The long-term thermal stability of DrUox. The enzyme preincubated at 25 °C (black triangles), 37 °C (black squares), 55 °C (black circles), and 70 °C (inverted black triangles) for each time point (1, 2, 3, 4, 5, 6, 12 and 24 h) were collected and the residual activity was assayed under the optimal condition.

To evaluate the long-term thermal stability of DrUox, the enzyme was preincubated for a long period, and then the residual activity of DrUox was assessed under the optimal condition. As shown in Figure 5, DrUox retained almost 100% of the activity at 25 °C and 30 °C for 24 h, whereas the retained activity decreased significantly when the preincubation temperature was >55 °C. After 1-h preincubation at 55 °C, only 10% residual activity remained. Therefore, DrUox exhibits high thermal stability at mesophilic conditions, which suggests its potential role in drug development.

### 2.6. Analysis of Structural Properties of DrUox

First, we attempted to estimate the presence of a regular secondary structure in DrUox, which can be recognized from the wavelengths of peaks in the circular dichroism spectra. The far-UV CD spectrum of DrUox showed a negative ellipticity peak at 216 nm with a positive ellipticity peak detected at 196 nm. Accordingly, Supplementary Figure S5 shows the spectra of recombinant DrUox, which is rich in alpha/beta structures. CDPro [32] deconvolution of the spectrum confirmed the visual assessment and pointed toward an α-helix-β-sheet protein secondary structure (25% α-helix, 27% β-sheet, 20% turn and 28% unordered), which is similar to other published Uox structural data.

**Figure 6:**
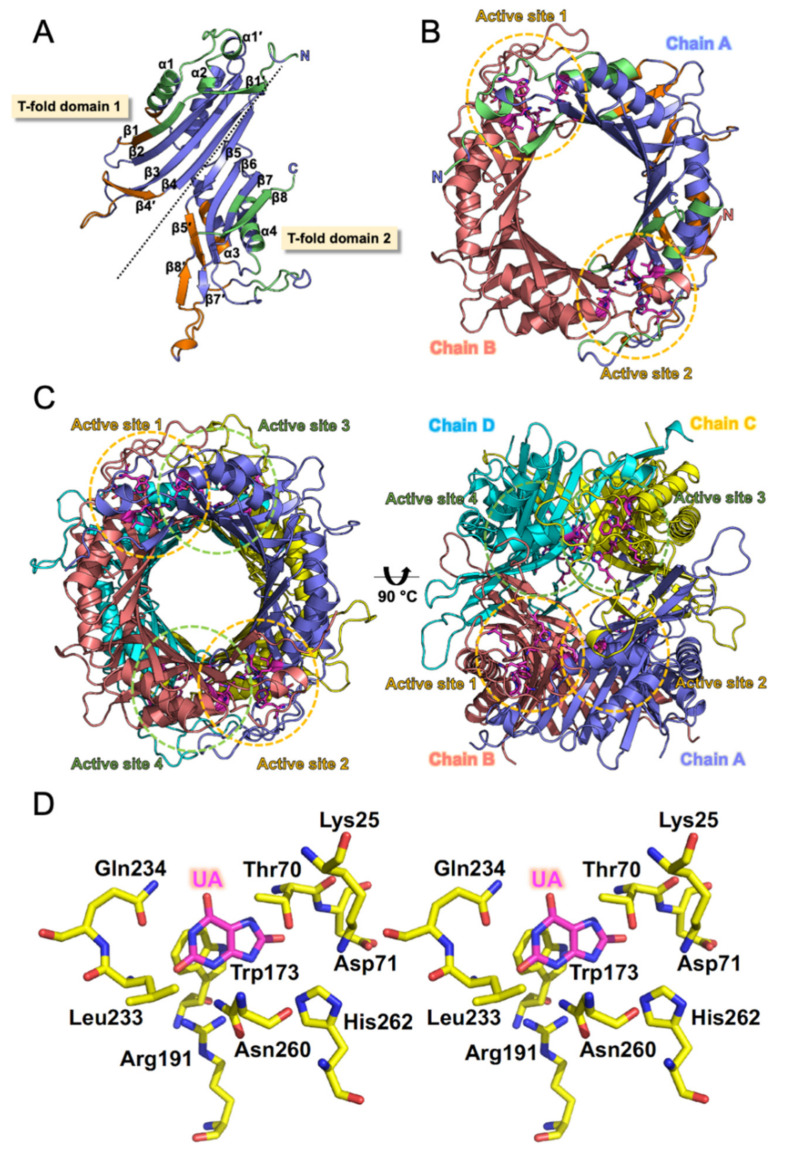
The structural model of DrUox. (**A**) Overall structure of the protomeric DrUox model. Structure of DrUox is represented by a cartoon model with α-helices, β-strands and loops in slate, and the secondary structures of DrUox are in black. The regions depicted in green and orange indicate the dimeric and tetrameric interfaces, respectively. (**B**) Top view of the dimeric DrUox. Chain A and Chain B of DrUox are depicted in slate and salmon, respectively. Two active sites within the dimeric DrUox are highlighted in the circle with dotted lines (orange). Active site residues are shown as sticks, with carbon in magenta. (**C**) Left, top view of the tetrameric DrUox. Right, side view of the tetrameric DrUox. Chain A, Chain B, Chain C, and Chain D of DrUox are depicted in slate, salmon, yellow, and cyan, respectively. Four active sites within the tetrameric DrUox are highlighted in the circle with dotted lines (orange and green). (**D**) A stereoview of the active site from the DrUox-UA model. Amino acids contributed by the dimeric DrUox are shown as sticks with carbon in yellow and labeled in black. Oxygen and nitrogen are in red and blue, respectively. Uric acid (UA) is shown as sticks, with carbon in magenta.

To understand the structural characteristics of DrUox, we performed crystallization of the recombinant DrUox. The protein crystal was obtained to diffract at low resolution with high mosacity (Supplementary Figure S6). We attempted several times to improve the crystal quality but were not successful. Therefore, molecular modeling of DrUox was used with the AgUox structure (PDB: 2YZC) as a template. Then structural quality, structural stability, and accuracy of DrUox model were further investigated by various tools (Supplementary Table S2). The final structural model of DrUox was built as four identical subunits according to the biochemical evidence from SEC data (Figure 2B). Structure of the DrUox protomer and its secondary structure elements are shown in Figure 6A. Each protomer contained a sequential eight-stranded β-sheet (β1–β8), five short β-strands (β1′, β4′, β5′, and β7′–β8′), four main α-helices (α1–α4), and a one-turn α-helix (α1′). Sequential β-strands packed into a curved β-sheet along with the four α-helices laid on the concave site of the sheet. The protomer can be divided into two structurally equivalent domains known as tunneling-fold (T-fold) domains [1] and exhibited antiparallel superfold ββααββ topology of each (T-fold domain 1 and 2). Moreover, the active site of the DrUox protomer may be surrounded by the β5–β6 loop, α4 helix, and β8-strand as described [33].

As shown in Figure 6B, two monomeric subunits (Chain A and B) are related by a crystallographic two-fold symmetry axis and are assembled as a dimer because of tight interactions. The dimer interface and tunnel formation contain two major regions, one formed by β1, β1′, α1, and α1′ from T-fold domain 1 and the other formed by β7, β8, α4, the β5–β6 loop, and the C-terminal loop from T-fold domain 2 (Figure 6A,B). The functional unit of the DrUox tetrameric structure is formed by the oligomerization of pairwise dimers (Chain A + B and Chain C + D; Figure 6C). The tetrameric interface region from the stacked dimers consists of parts of β1, α1, and β4′ from T-fold domain 1 and α3, β5′, β8′, and the β7′–β8′ loop from T-fold domain 2 (Figure 6A). Notably, the C-terminal loop present in the previous reported thermostable urate oxidases [20] was absent in DrUox. However, DrUox still exhibited thermal stability under mesophilic conditions. Altogether, the tetrameric conformation of DrUox may be stabilized by a combination of dimeric and tetrameric interface interactions.

## 3. Discussion

Previous steady-state kinetic studies indicate that an ionizable group on the Uox enzyme with a pKa of 6.4 must be unprotonated for catalysis. It is proposed that a novel Thr-Lys catalytic diad (Thr70 and Lys25 in DrUox) acts as the general base to abstract a proton from the N9 position of the substrate to generate the dianion [34,35]. Interestingly, voltammetric and quantum mechanical studies of urate oxidation in aqueous solution have shown that the reaction becomes more facile with increasing pH [36,37]. During the reaction, the best substrate reactant for dioxygen is urate dianion because all urate dianions have negative ionization potentials. They trend to transfer spontaneously one of their electrons to dioxygen, while neutral uric acid and urate monoanions do not. Since uric acid has two pKa values of 5.4 and 9.8 [38], urate dianion forms are mainly present in high pH solution. This is a reason why urate oxidases reach their maximum activity between pH 8 and pH 10 generally [39,40].

Thermal stability of urate oxidase is an emerging challenge for protein drug development. Strategies including genome mining, rational design, and directed evolution have been widely used to improve protein thermal stability. However, only several thermostable urate oxidases have been studied. Here, we reveal a thermostable urate oxidase of DrUox in biochemical aspects. DrUox possesses high catalytic activity and thermal stability at moderate temperatures, thus providing a potential target for drug development. To further evaluate the thermal stability of DrUox, we compared DrUox with other thermostable urate oxidases from various microorganisms (Table 2). Some urate oxidases with high specific activity are not so thermostable. The urate oxidase from *A. flavus* retains only 30% activity after incubation at 40 °C for 1 h; its specific activity is 27 U mg^−1^ [14,41]. The urate oxidase from *C. utilis* retains only 40% activity after incubation at 37 °C for 24 h; its specific activity is 38.4 U mg^−1^ [27]. However, some urate oxidases possess thermostability with unspectacular specific activity. A urate oxidase from *B. firmus* DWD-33 exhibits higher thermostability that retains almost 100% activity after incubation at 60 °C for 1 h, whereas its specific activity is only 9.58 U mg^−1^ [42]. A urate oxidase from *Microbacterium* sp. strain ZZJ4-1 retains almost 100% activity after incubation at 65 °C for 0.5 h, but its specific activity is only 5.32 U mg^−1^ [43]. Nevertheless, a urate oxidase from *K. marixianus* possesses high specific activity and thermostability. It retains 79% activity after incubation at 40 °C for 90 h and its specific activity is 50.54 U mg^−1^ [28]. In the present study, the specific activity of DrUox was 38.06 U mg^−1^, which is rather high relative to the reported urate oxidases. Furthermore, it retains almost 100% activity after incubation at 37 °C for 24 h, which suggests that DrUox is a thermostable enzyme.

To interpret the catalytic roles within the DrUox active site, we build a DrUox-UA model using AgUox-UA (PDB code: 2YZB) as a template (Figure 6D). Similar to previous studies, the active sites of DrUox consist of pairwise subunits (Chain A + B or Chain C + D) and exhibit a positively charged cavity. One subunit contributes residues from *α*4 and the β5–β6 loop, whereas the other contributes residues from β1 and α1. Moreover, because of the high conservation of the catalytic residues within the active site of DrUox, its catalytic mechanism can be inferred. A water molecule may be located above the UA that is held tightly by the side-chains of Asn260 from Chain A and Thr70 from Chain B via H-bonds, which may act as reagent tweezers as previously described [33,44,45]. His262 from Chain A combined with Lys25 and Thr70 from Chain B may serve as an important catalytic triad for proton transfer [33,35,46], whereas the other water molecule may play a role of proton shuttle during the reaction [35].

Urate oxidases have been developed as protein drugs for treating hyperuricemia and TLS. However, the protein instability and immunogenicity affect the effectiveness of these therapeutic agents. Hence, thermal stability is a key issue for protein drugs. We reveal a thermostable urate oxidase from *D. radiodurans* in terms of its biochemical aspects. The tetrameric DrUox demonstrated high catalytic efficiency and thermal stability under mesophilic conditions. Structural analysis of DrUox confirmed the catalytic mechanism on the basis of the conserved catalytic residues. This study reveals the biochemical basis of a thermostable urate oxidase with high catalytic efficiency and thermal stability, thus providing a potential target for drug development.

## 4. Materials and Methods

### 4.1. Materials

PrimeSTAR HS DNA polymerase was purchased from Takara Bio (Otsu, Kusatsu, Japan). Restriction endonucleases and T4 DNA ligase were from Thermo Fisher Science (Hudson, NH, USA). PrimeSTAR HS DNA polymerase was from Takara Bio (Otsu, Kusatsu, Japan). Kanamycin, isopropyl β-_D_-1-thiogalactopyranoside (IPTG), and all media supplements (MDBio, Inc., Taipei, Taiwan) were used for bacterial culture. The *Escherichia coli* strain BL21 (DE3) and the pET-28a vector (Novagen, Madison, WI, USA) were used for protein expression. Uric acid sodium salt, all other chemicals and reagents were from Sigma-Aldrich (St. Louis, MO, USA).

### 4.2. Genomic DNA Extraction

*Deinococcus radiodurans R1* was acquired from the Bioresource Collection and Research Center (Hsinchu, Taiwan). A 5-mL amount of *Deinococcus radiodurans* (OD_600_ 2.0) was cultured with GPHF medium at 55 °C for 3 days and pelleted by centrifugation at 6000 rpm for 10 min. The pellets were resuspended with 250 μL TE buffer (50 mM Tris-HCl, pH 8.0, 50 mM EDTA) supplemented with 1 mg/mL lysozyme. The lysates were placed on ice for 60 min followed by the addition of 90 μL STEP buffer (0.5% sodium dodecyl sulfate [SDS], 50 mM Tris, pH 8.0, 40 mM EDTA, 2 mg/mL proteinase K) for another 60 min. An amount of 90 μL NH_4_OAc and 500 μL phenol/chloroform was added to the lysates, followed by centrifugation at 6000× *g* for 10 min. The aqueous layer was then collected, followed by the addition of 500 μL chloroform/isoamyl alcohol for centrifugation at 6000× *g* for 10 min. The aqueous layer was collected, followed by the addition of 0.6-fold volume of isopropanol and cooling at −80 °C for 30 min. The solution was centrifuged at 800× *g* for 5 min, and the supernatant was discarded. The precipitated pellet was washed with 70% ethanol three times and air-dried. The genomic DNA was finally dissolved in sterile water and stored at −20 °C.

### 4.3. Plasmid Constructs, Protein Expression and Purification

The urate oxidase gene (*Uox*, CP001874.1) was amplified from the genomic DNA of *Deinococcus radiodurans* (R1) with the primers for DrUox-F (5′-CGGCATATGACGGGAACCCAGCAACCG-3′ containing *Nde*I site) and DrUox-R (5′-AATCTCGAGTCACTCGGCGCGCTCCAC-3′ containing *Xho*I site) by using high-fidelity thermostable PrimeSTAR HS DNA polymerase (Takara Bio Inc., CA, USA). The PCR product (897-bp DNA fragment) was inserted into the corresponding restriction sites of the pET-28a vector system (Novagen, WI, USA). The expression plasmid of DrUox was transformed into *E. coli* BL21(DE3) cells by growing them in 1 L Luria-Bertani (LB) medium with 50 μg/mL kanamycin until reaching OD_600_ 0.8 at 37 °C and induced with the addition of 0.1 mM IPTG for 20 h at 25 °C. Cells were harvested by centrifugation at 6000 rpm and lysed in lysis buffer (20 mM Tris-HCl pH 8.0, 100 mM NaCl, 1 mM phenylmethanesulfonyl fluoride) by sonication on ice. Lysates were clarified by centrifugation at 13000 rpm for 20 min and filtered through a 0.45-mm filter membrane to remove debris, then applied to the column containing Ni^2+^-nitrilotriacetic acid (Ni^2+^-NTA) resin. His-tagged DrUox was eluted by lysis buffer containing 100 mM imidazole, then the protein fractions were dialyzed against stabilization buffer (20 mM Tris-HCl pH 8.0, 100 mM NaCl, 1 mM dithiothreitol [DTT]). After the removal of the poly-histidine fusion tag from recombinant protein by thrombin protease, the tag-free recombinant protein was concentrated in Amicon Ultra centrifugal filters (Merck Millipore, MA, USA) and quantified by UV_280_.

### 4.4. Molecular Characterization by SDS-PAGE and Size Exclusion Chromatography

Concentrated DrUox was resolved by 12% SDS-PAGE to determine the molecular weight in denatured state. To determine the oligomeric states of DrUox, the protein sample was applied to gel filtration chromatography with a Superdex 200 (10/300) GL column (GE Healthcare, WI, USA) equilibrated with Tris-HCl buffer (20 mM Tris hydrochloride buffer pH 8.0 with 100 mM NaCl) at a flow rate of 0.5 mL/min. The standard mixture was also injected onto the FPLC system with the same buffer condition. Consequently, an apparent peak of eluted DrUox at the indicated elution volume was calculated by interpolation between the calibration lines, and its oligomeric state in solution was determined.

### 4.5. Circular Dichroism (CD) Spectroscopy

Far-UV CD spectra were recorded on a Jasco J-810 spectropolarimeter (Jasco International Co., Tokyo, Japan). CD measurements were carried out in a 1-mm quartz cuvette at wavelength 190 to 260 nm. The protein sample in phosphate buffer was set to 10 μM at pH 7.0 and centrifuged at 10,000× *g* for 10 min before analysis. Baseline subtraction, smoothing and data normalization involved use of SigmaPlot. The CD data are shown as mean residue ellipticity units (deg cm^2^ dmol^−1^).

### 4.6. Thermal Shift Assay

The thermostability of the DrUox was evaluated by a thermal shift assay with DSF as described previously. In brief, a 25-μL mixture containing 2 μL SYPRO Orange (Sigma-Aldrich, MO, USA), 1.25 μL dialysis buffer (20 mm Tris-HCl, and 100 mm NaCl, pH 7.0), and 10 μL of 1 μM protein sample was mixed on ice. All reactions were performed in triplicate in an 8-well PCR tube. Fluorescent signals were measured using a CFX48 Real-Time PCR Detection System (BioRad, CA, USA) with a temperature range from 25 to 95 °C in 0.1 °C/30-s steps (excitation, 450–490 nm; detection, 560–580 nm). Data evaluation and Tm determination involved use of the Bio-Rad CFX Manager.

### 4.7. Urate Oxidase Activity Assay

Urate oxidase activity was assayed by monitoring the depletion of uric acid, which was detected by a decrease in absorbance at 293 nm [20,26,47]. The optimal temperature of DrUox was evaluated by incubating 0.5 μM DrUox and 500 μM UA in a temperature range from 10 to 80 °C for 3 min in pH 9.0, then the residual activity was assayed. The optimal pH of DrUox was evaluated by incubating 0.5 μM DrUox and 500 μM UA in a pH range of 3.0 to 11.0 (at 30 °C for 3 min, then the residual activity was assayed. The pH buffers comprised Na_2_HPO_4_-Citrate (pH 3.0–7.0) and Glycine-NaOH (pH 8.0–11.0). Temperature stability of DrUox was determined under optimal conditions after preincubating DrUox for 0.5 h at different temperatures ranging from 10 °C to 80 °C. pH stability of DrUox was determined under optimal conditions after preincubating DrUox for 24 h at 4 °C in pH buffers ranging from 3.0 to 11.0. The kinetic parameters were determined using 0.1 mg of DrUox with different concentrations (200–600 μM) of UA for 3 min under optimal (0.1 M Glycine-NaOH buffer pH 9.0 at 30 °C) and physiological conditions (0.1 M Na_2_HPO_4_-Citrate buffer pH 7.4 at 37 °C). The reaction was stopped by the addition of 1% KOH. Molar extinction coefficient for UA was assumed as 1.26 × 10^4^ M^−1^∙cm^−1^. The values of *K*_m_ and *K*_cat_ for the DrUox were calculated by nonlinear regression with Prism 8 (GraphPad software, CA, USA) and the Michaelis-Menten plots were shown in Supplementary Figure S3.

### 4.8. Effect of Metal Ions, Surfactants, Chelating Agent, Reducing Agents and Oxidative Agent

To evaluate the effect of the metal ions on DrUox, the purified enzyme was preincubated at 4 °C for 1 h with the following metal ions: 2 mM each of KCl, CaCl_2_, CoCl_2_, CuSO_4_, MgSO_4_, MnCl_2_, NiSO_4_, FeSO_4_ and Fe_2_(SO_4_)_3_. To assess the effect of the chemical agents, the enzyme was preincubated with 10 and 20 mM of EDTA, DTT, β-ME; 1% and 2% of Triton X-100 and Tween-20; and 0.2 mM and 0.5 mM of H_2_O_2_. The enzymatic activity was measured under the optimal condition. The activity without treatment was designated as 100%. Each assay under each condition was performed in triplicate.

### 4.9. Long-Term Thermal Stability Measurement

Temperature tolerance of DrUox was assessed by monitoring the depletion of UA at the indicated temperatures and times. The enzyme was incubated at 25, 37, 55 and 70 °C for each time (1, 2, 3, 4, 5, 6, 12 and 24 h), and the residual activity was assayed during the incubation periods. The protein stability assays at each condition were performed in triplicate.

### 4.10. Homology Modeling and Quality Evaluation of DrUox

The structure model of DrUox was built by using the SWISS-MODEL server [48] and refined by using the GalaxyWEB server [49]. According to the SWISS-MODEL template library search, a total of 36 templates was found. The DrUox model was generated based on the top template, *A. globiformis* Uox (PDB: 2YZC), which shares 43.0% sequence identity. The structural quality, structural stability, and accuracy of DrUox model were further investigated by various tools. The QMEAN Z-score of the predicted protein model is −2.37, which means good quality structure. The stereochemical properties of the DrUox model is assessed by ProQ [50] and Verify3D [51]. The DrUox model showed a ProQ-LG scrore of 4.25, which suggests an extremely good model. Analysis with Verify3D revealed that >79% of the residues in the model had a 3D-1D score >0.2, which indicates that most residues are in the favorable region (Supplementary Figure S7A). Overall quality of the DrUox model was assessed by ProSA [52], PROCHECK [53], and ERRAT [54]. The ProSA Z-score of −5.15 is within the range of experimentally determined protein structures (Supplementary Figure S7B). The Ramachandran Plot was generated via PROCHECK [53]. The DrUox model showed 89.3% of the amino acid residues in the most favoured region and the other 10.7% of the residues were present in the allowed region (Supplementary Figure S7C). ERRAT is a so-called overall quality factor for analyzing the statistics of non-bonded interactions between different atom types [54]. The overall quality factor predicted by the ERRAT server was 88.47 (Supplementary Figure S7D). Consequently, the DrUox model is well established for further structural studies. The 3D structural visualization was performed on PyMOL (http://www.pymol.org (accessed on 4 August 2014)).

## Figures and Tables

**Table 1:** Steady-state kinetic parameters of DrUox under physiological and optimal conditions.

pH	Temperature (°C)	*K*_m_ (μM)	*K*_cat_ (s^−1^)	*K*_cat_/*K*_m_ (s^−1^∙μM^−1^)
7.4	37	760.81 ± 85.89	1.41 ± 0.12	0.20 × 10^−2^ ± 0.01
9.0	30	332.58 ± 57.94	17.49 ± 1.49	5.35 × 10^−2^ ± 0.01

**Table 2:** Urate oxidase from various microorganisms.

Original Microbes	Genbank Accession No.	Expression Hosts	Specific Activity (U mg^−1^)	Optimal Temperature (°C)	Temperature Tolerance
*D. radiodurans* [this study]	AAF10733	*E. coli*	38.06	30	37 °C, 24 h, 100%
*A. flavus* [14,41]	CAA43895	*E. coli*	27	30–37	40 °C, 1 h, 30%
*B. firmus* DWD-33 [42]	—	—	9.58	50	60 °C, 1 h, 100%
*C. utilis* [27]	P78609	*E. coli*	38.4	37	37 °C, 24 h, 40%
*K. marixianus* [28]	BAP70065	*E. coli*	50.54	42	40 °C, 90 h, 79%
*Microbacterium* sp. strain ZZJ4-1 [43]	AEY68606	—	5.32	30	65 °C, 0.5 h, 100%

## Data Availability

The data presented in this study are available in article.

## Supplementary Materials

The following are available online at https://www.mdpi.com/article/10.3390/ijms22115611/s1, Figure S1: DrUox gene amplified from the genomic DNA of *Deinococcus radiodurans* R1, Figure S2: Protein expression of DrUox, Figure S3: Michaelis-Menten plots of DrUox Kinetic assay, Figure S4: Validation of structural effects on DrUox in the presence of metal ions. Figure S5: Circular dichroism (CD) spectra of DrUox. Figure S6: An image of the rod-shaped and plate-like DrUox crystals in complex with uric acid and its protein diffraction pattern, Figure S7: Evaluation of the predicted DrUox model. Table S1: Effect of various metal ions and chemical agents on the enzyme activity of DrUox. Table S2: Assessment of the predicted structure model of DrUox.
