# Supplementary material for: Molecular Elucidation of a Urate Oxidase from Deinococcus radiodurans for Hyperuricemia and Gout Therapy
Source: Int J Mol Sci. 2021 May 25;22(11):5611. doi: 10.3390/ijms22115611 (PMC8199477; doi:10.3390/ijms22115611)
Supplement: Supplementary file 1 [file ijms-22-05611-s001.zip › ijms-1183323-supplementary.pdf]

Molecular Elucidation of a Urate Oxidase from *Deinococcus radiodurans* for Hyperuricemia and Gout Therapy

Yi-Chih Chiu[1,2], Ting-Syuan Hsu[3#$], Chen-Yu Huang[3#&] and Chun-Hua Hsu[1,2,4*]

[1]Genome and Systems Biology Degree Program, National Taiwan University and Academia Sinica, Taipei, Taiwan.

[2]Department of Agricultural Chemistry, National Taiwan University, Taipei, Taiwan.

[3]Taipei First Girl High School, Taipei, Taiwan.

[4]Institute of Biochemical Sciences, National Taiwan University, Taipei, Taiwan.

#These authors contributed equally and are listed in alphabetical order.
$Current address: School of Medicine, National Taiwan University, Taipei, Taiwan
&Current address: Department of Electrical Engineering, National Taiwan University, Taipei, Taiwan

*Corresponding author: Dr. Chun-Hua Hsu

E-mail: andyhsu@ntu.edu.tw, Contact information: +886-2-33664468

A

Oligonucleotide primers used in the study.

Name	Sequence: 5′ – 3′
Cloning for recombinant protein expression	
DrUox-F	CGGCATATGACGGGAACCCAGCAACCG
DrUox-R	AATCTCGAGTCACTCGGCGCGCTCCAC

B

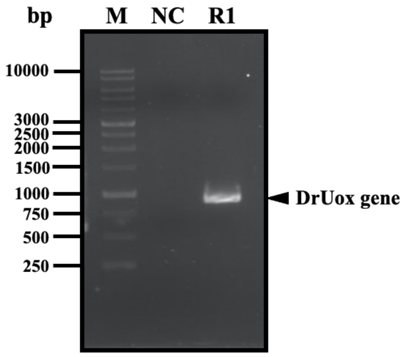

Supplementary Figure S1. *DrUox* gene amplified from the genomic DNA of *Deinococcus radiodurans R1*. (A) Oligonucleotide primers used in the study. (B) *DrUox* gene (897 bp) was amplified from the gDNA of *Deinococcus radiodurans R1* using the primer pair DrUox-F and DrUox-R. Black arrow indicates the PCR product of *DrUox* gene. NC: negative control.

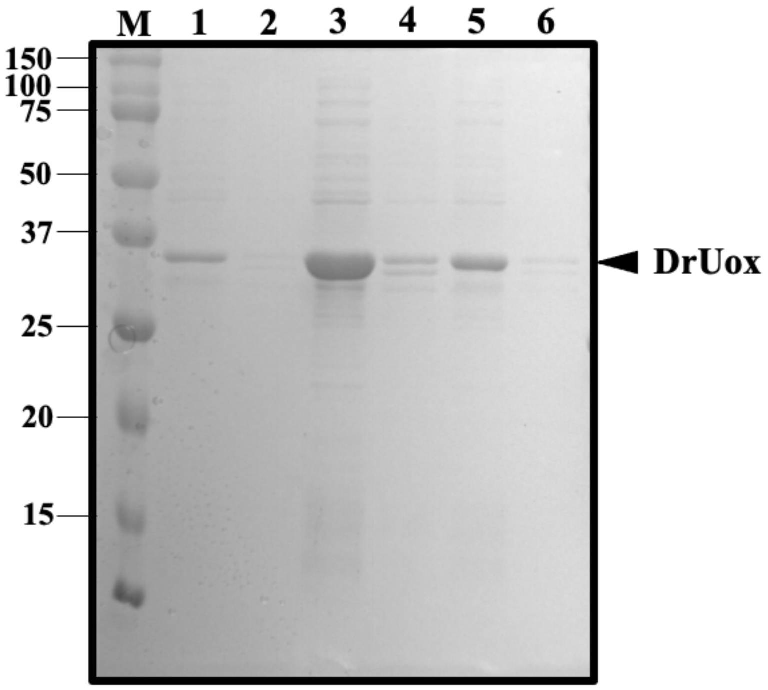

Supplementary Figure S2. Protein expression of DrUox. Lane 1: Supernatant of non-induction control; Lane 2: Pellet of non-induction control; Lane 3: Supernatant of 0.1 mM IPTG induction; Lane 4: Pellet of 0.1 mM IPTG induction; Lane 5: Supernatant of 1 mM IPTG induction; Lane 6: Pellet of 1 mM IPTG induction. Black arrow indicates the induced expression of DrUox.

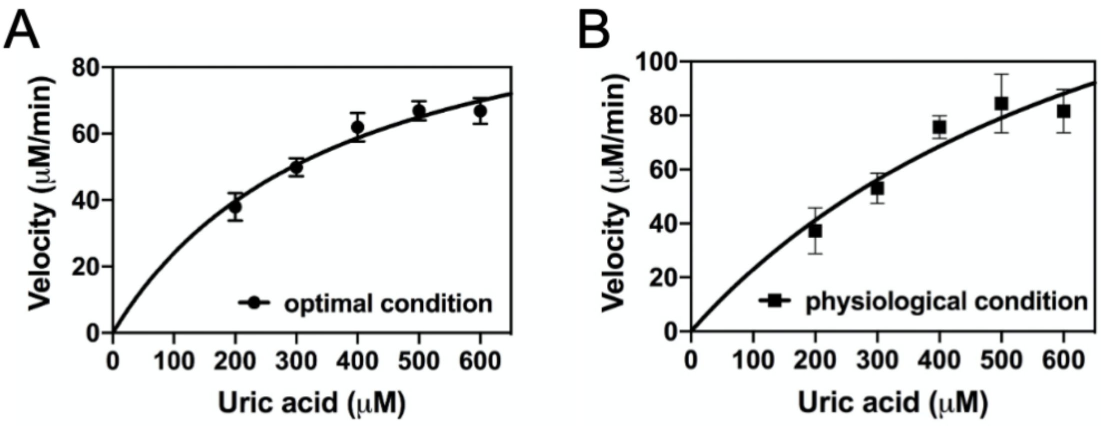

Supplementary Figure S3. Steady-state kinetics of DrUox under optimal and physiological conditions. DrUox activity under (A) optimal (30 °C, pH 9.0) and (B) physiological (37 °C, pH 7.4) conditions were determined by rate of reaction (μM x min^{-1}) that express rate of reduction uric acid concentration in time unit and were measured by kinetic mode (see Materials and methods). Solid lines represent Michaelis-Menten kinetic fits performed in Prism 8. These data are represented as mean ± SEM (n = 3 independent experiments). Kinetic parameters are shown in Table 1.

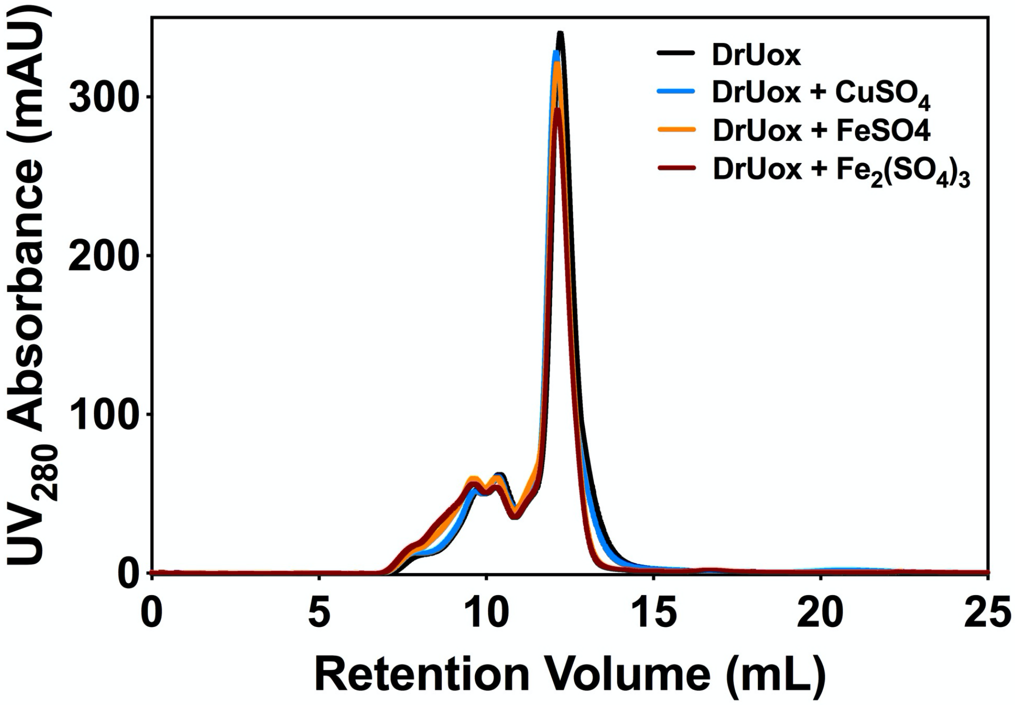

Supplementary Figure S4. Validation of structural effects on DrUox in the presence of metal ions. The native DrUox was preincubated for 1 h in the presence of the following salts: 2 mM each of $CuSO_4$ (cyan line), $FeSO_4$ (orange line), and $Fe_2(SO_4)_3$ (brown line), and analyzed by size-exclusion chromatography. DrUox in the absence of metal ions (black line) was a control.

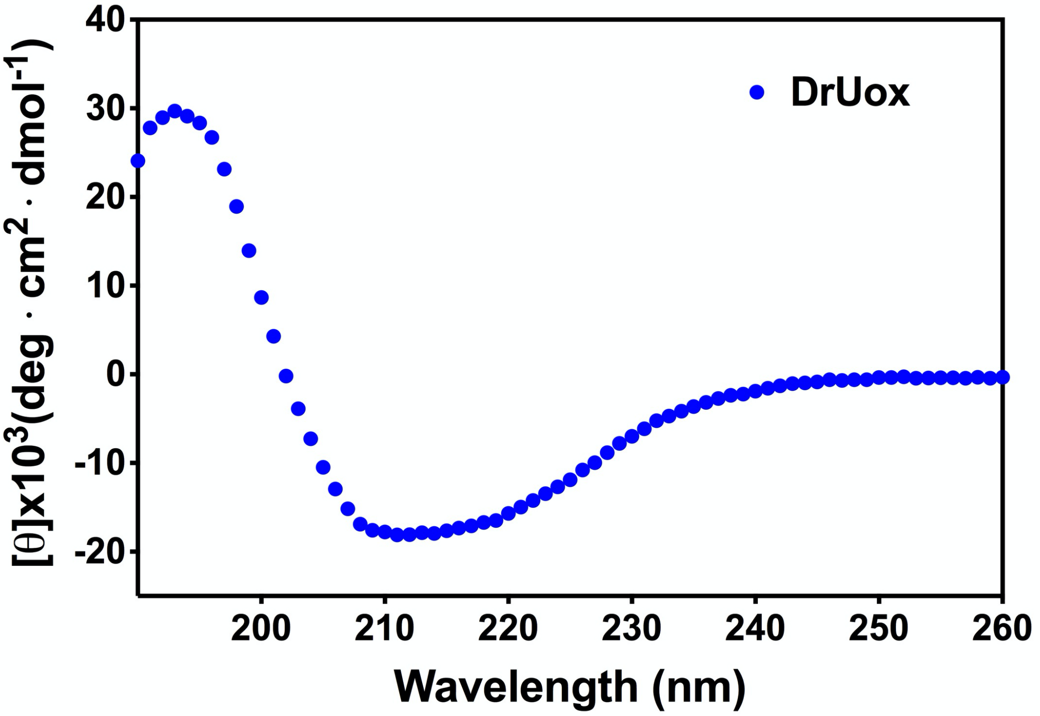

Supplementary Figure S5. Circular dichroism (CD) spectra of DrUox. The CD spectra were recorded at 25 °C with 10 μM DrUox in CD buffer (20 mM phosphate buffer, pH 7.0) from 260 to 190 nm.

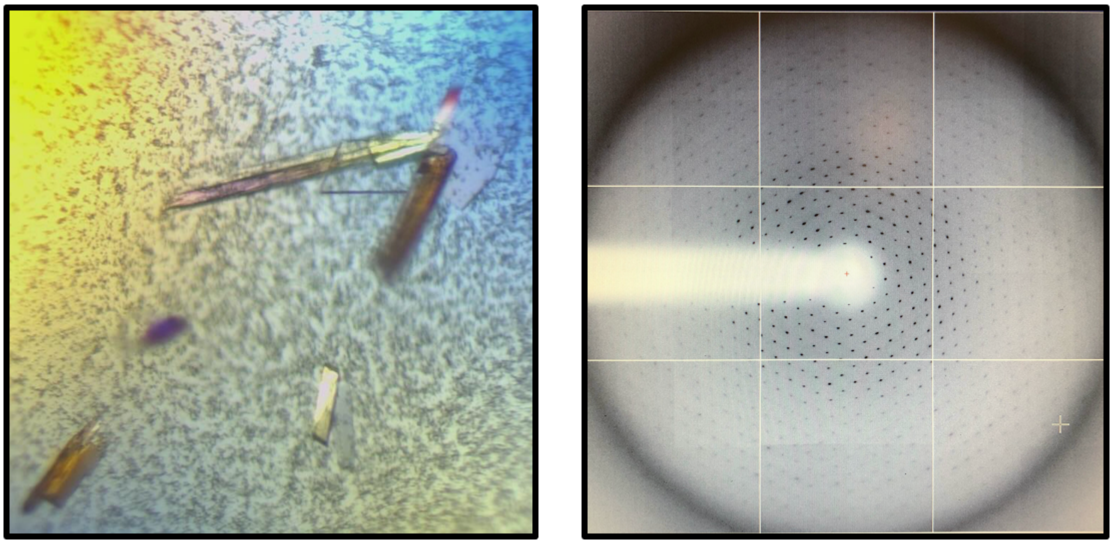

Supplementary Figure S6. An image of the rod-shaped and plate-like DrUox crystals in complex with uric acid and its protein diffraction pattern. (A) Recombinant DrUox was mixed with uric acid in a 1:20 protein:substrate ratio. Crystallization in 0.02 M sodium/potassium phosphate, 0.1 M Bis-Tris propane, pH 7.5, and 20% PEG 3350 using the uric acid-containing protein solution yielded blue needles and clusters of small blue crystals. (B) The high-resolution diffraction pattern from the DrUox-UA crystals.

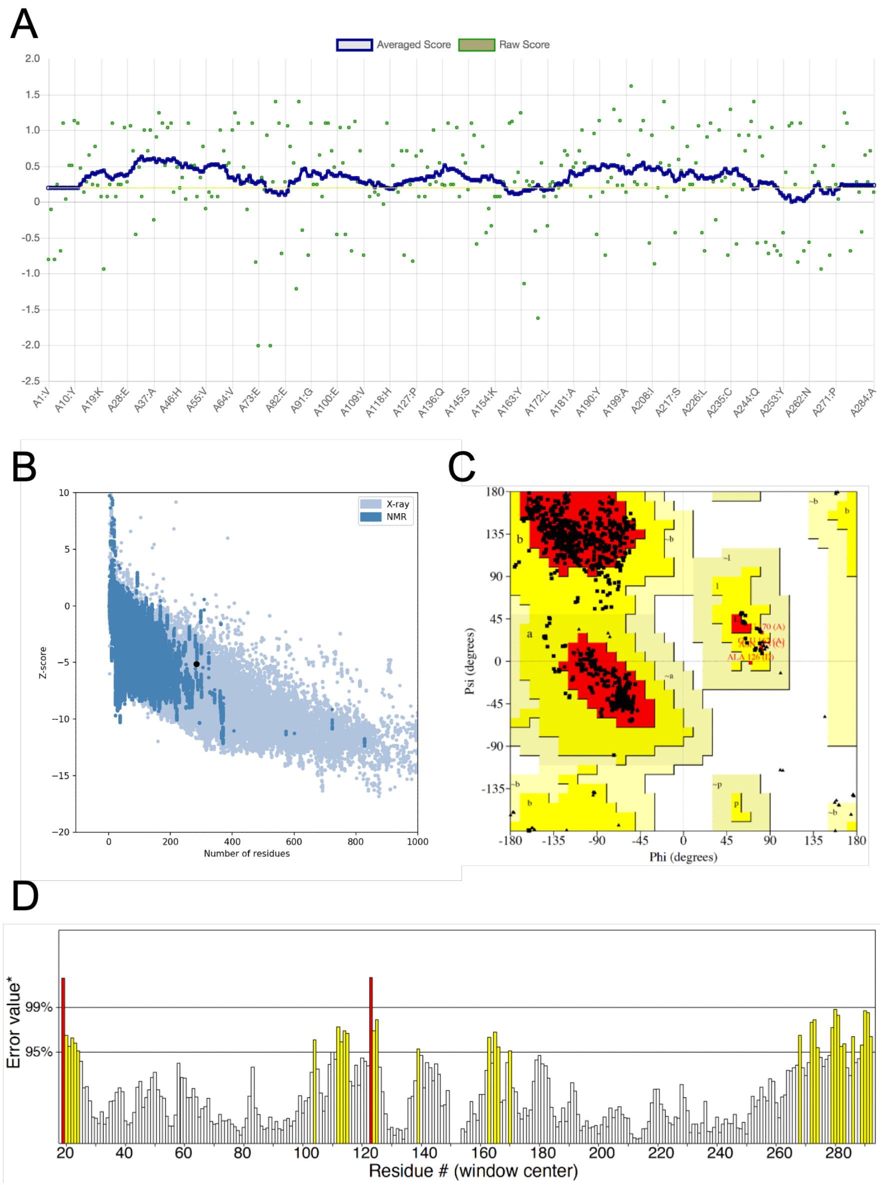

Supplementary Figure S7. Evaluation of the predicted DrUox model. (A) Validation of the DrUox model by Verify3D. The residues with a mean 3D-1D score > 0.2 are considered reliable. (B) ProSA Z-score plot of the DrUox model. ProSA Z-score plot shows Z-score (black dot) in a plot that contains the Z-scores of all experimentally determined protein chains currently available in the Protein Data Bank. Blue color region represents Z-scores of protein structures characterized by NMR analysis and grey color region represents Z-scores of protein structures characterized by X-ray diffraction studies. (C) Ramachandran plot of the DrUox model. Ramachandran plot shows residues in most favoured regions (red), additional allowed regions (yellow), generously allowed regions (light yellow) and disallowed regions (white). (D) Analysis of the ERRAT result. In ERRAT plot, two black lines show regions of the 3D model that can be rejected at the 95% and 99% confidence level.

Supplementary Table S1. Effect of various metal ions and chemical agents on the enzyme activity of DrUox.

**Metal ions or chemicals**	**Concentration**	**Relative activity (%)**
Control	−	100.0
KCl	2 mM	96.32 ± 1.61
$CaCl_2$	2 mM	86.49 ± 1.10
$CoCl_2$	2 mM	91.89 ± 1.19
$CuSO_4$	2 mM	36.54 ± 0.73
$MgSO_4$	2 mM	121.40 ± 0.71
$MnCl_2$	2 mM	85.03 ± 2.87
$NiSO_4$	2 mM	88.23 ± 0.88
$FeSO_4$	2 mM	42.24 ± 0.83
$Fe_2(SO_4)_3$	2 mM	22.99 ± 0.83
EDTA	10 mM	89.89 ± 2.82
EDTA	20 mM	84.16 ± 1.33
Triton X-100	1 %	97.72 ± 2.66
Triton X-100	2 %	82.38 ± 4.32
Tween-20	1 %	97.7 ± 2.97
Tween-20	2 %	103.43 ± 1.98
DTT	10 mM	90.84 ± 0.90
DTT	20 mM	90.77 ± 0.99
β-ME	10 mM	92.50 ± 0.32
β-ME	20 mM	91.71 ± 1.13
H_2O_2	0.2 mM	99.14 ± 1.64
H_2O_2	0.5 mM	99.48 ± 6.57

Supplementary Table S2. Assessment of the predicted structure model of DrUox.

**Validation Index**	
SWISS-MODEL<br>GMQE<br>QMEAN	 0.76<br>-2.37
Ramachandran plot (PROCHECK)<br>Residues in most of favoured regions<br>Residues in additional allowed regions<br>Residues in generously allowed regions<br>Residues in disallowed regions	 89.3%<br>10.3%<br>0.4%<br>0.0%
ProSA Z-score	-5.15
ERRAT score	88.47
Verify3D (% of amino acids with average 3D-1D score ≥ 0.2)	79.14%
